# Mutations of EXOSC3/Rrp40p associated with neurological diseases impact ribosomal RNA processing functions of the exosome in *S. cerevisiae*

**DOI:** 10.1261/rna.060004.116

**Published:** 2017-04

**Authors:** Abby Gillespie, Jason Gabunilas, Joanna C. Jen, Guillaume F. Chanfreau

**Affiliations:** 1 Department of Chemistry and Biochemistry and the Molecular Biology Institute, University of California, Los Angeles, California 90095-1569, USA; 2 Department of Neurology, University of California, Los Angeles, California 90095-1763, USA; 3 Department of Neurobiology, University of California, Los Angeles, California 90095-1763, USA

**Keywords:** pontocerebellar hypoplasia, exosome, EXOSC3, Rrp40, ribosomal RNA processing

## Abstract

The RNA exosome is a conserved multiprotein complex that achieves a large number of processive and degradative functions in eukaryotic cells. Recently, mutations have been mapped to the gene encoding one of the subunits of the exosome, EXOSC3 (yeast Rrp40p), which results in pontocerebellar hypoplasia with motor neuron degeneration in human patients. However, the molecular impact of these mutations in the pathology of these diseases is not well understood. To investigate the molecular consequences of mutations in *EXOSC3* that lead to neurological diseases, we analyzed the effect of three of the mutations that affect conserved residues of EXOSC3/Rrp40p (G31A, G191C, and W238R; G8A, G148C, and W195R, respectively, in human and yeast) in *S. cerevisiae*. We show that the severity of the phenotypes of these mutations in yeast correlate with that of the disease in human patients, with the W195R mutant showing the strongest growth and RNA processing phenotypes. Furthermore, we show that these mutations affect more severely pre-ribosomal RNA processing functions of the exosome rather than other nuclear processing or surveillance functions. These results suggest that delayed or defective pre-rRNA processing might be the primary defect responsible for the pathologies detected in patients with mutations affecting EXOSC3 function in residues conserved throughout eukaryotes.

## INTRODUCTION

The RNA exosome is a multiprotein complex first identified in *S. cerevisiae* (Mitchell et al. 1997) and conserved in eukaryotic cells (Allmang et al. 1999b). This complex exhibits 3′–5′ exoribonuclease activity, as well as endonuclease activity mediated by the Dis3p/Rrp44p subunit of the complex (Lebreton et al. 2008; Schaeffer et al. 2009; Schneider et al. 2009). The Dis3p/Rrp44p catalytic subunit associates with a core ring of six subunits and with three additional subunits to form a catalytically active complex (Wasmuth et al. 2014; Makino et al. 2015; Zinder et al. 2016). This complex is found both in the nucleus and cytoplasm of eukaryotic cells (Allmang et al. 1999a) and is involved in a variety of RNA processing and degradation functions. The major quantitative function of the exosome in eukaryotic cells is to process the precursor of ribosomal RNA by trimming some of the 3′-extensions of the pre-rRNAs that remain following some of the cleavage events (Allmang et al. 1999a). The nuclear exosome also processes the precursor species of various small nuclear and nucleolar RNAs to generate their mature 3′-ends (Allmang et al. 1999a). In addition to these processing functions, the nuclear exosome of *S. cerevisiae* also degrades a large number of cryptic transcripts or precursor species (Kadaba et al. 2004; Wyers et al. 2005; Gudipati et al. 2012) and is involved in the quality control of gene expression by degrading unspliced RNAs in the nucleus (Bousquet-Antonelli et al. 2000; Sayani and Chanfreau 2012). The cytoplasmic exosome has been implicated in a variety of degradative functions, including surveillance of transcripts that have escaped from the nucleus and are defective for translation termination (van Hoof et al. 2002).

**FIGURE 1.**
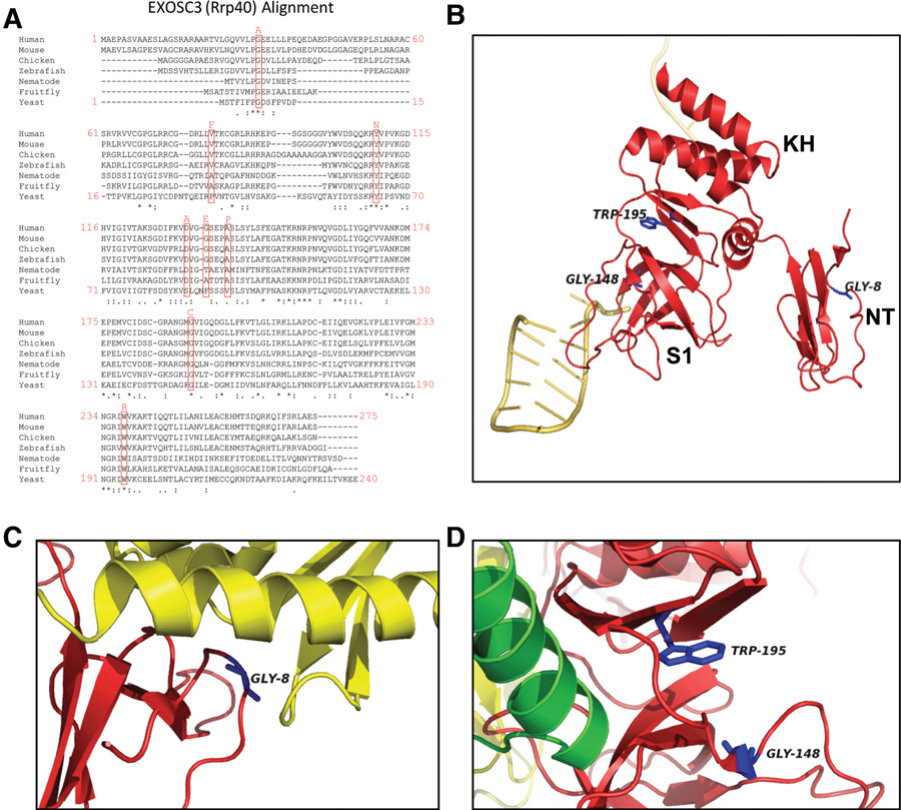
EXOSC3/Rrp40p mutations and location of the corresponding amino acids in Rrp40p structure. (*A*) Sequence alignment showing the position of mutations mapped in EXOSC3 and the conservation of the corresponding amino acids in eukaryotic EXOSC3 homologs. The positions of the amino acids are indicated at the beginning and end of each line for the human and yeast sequences. (*B*) Structure of Rrp40p in the context of the global structure of the exosome. The locations of the three residues analyzed and of the main protein domains are indicated. The yellow strand indicates the RNA substrate. Residues analyzed in this study are highlighted in blue. The figure was generated using PyMol from PDB entry 4IFD (Makino et al. 2013). (*C*) Close view of the atomic environment of Rrp40p Gly8. Rrp40p is shown in red; Rrp46p is shown in yellow. Gly8 is highlighted in blue. The figure was generated using PyMol from PDB entry 4IFD (Makino et al. 2013). (*D*) Close view of the atomic environment of Rrp40p Gly148 and Trp195. Rrp40p is shown in red. The green α helix shown is part of Rrp45p. Gly148 and Trp195 are highlighted in blue. The figure was generated using PyMol from PDB entry 4IFD (Makino et al. 2013).

In higher eukaryotes, RNA degradative functions of the exosome have started to emerge due to studies that have analyzed the phenotypes of cells depleted for various components of the exosome (Lubas et al. 2015; Pefanis et al. 2015). Recently, mutations affecting EXOSC3, the human homolog of Rrp40p, were shown to cause pontocerebellar hypoplasia type 1B (PCH1B, OMIM614678) (Wan et al. 2012). To date, biallelic *EXOSC3* mutations have been found in ∼50% of patients with PCH1, which is characterized by maldevelopment and degeneration of cerebellar and spinal motor neurons (Wan et al. 2012; Rudnik-Schöneborn et al. 2013; Eggens et al. 2014). Disease-causing mutations reside in various functional domains of EXOSC3, ranging from the putative KH RNA binding domain to the interface with the rest of the complex (Fig. 1). Different mutations appear correlated with different severity in clinical manifestations and survival (Wan et al. 2012; Rudnik-Schöneborn et al. 2013; Eggens et al. 2014). Thus, the precise impact of the mutations on the function of the human exosome remains enigmatic, and how these mutations lead to human disease remains unclear. To investigate the molecular impact of disease-causing mutations on the function of the exosome, we introduced several corresponding mutations into the Rrp40p subunit of *S. cerevisiae* and analyzed the RNA processing and degradation phenotypes of these mutants in vivo.

## RESULTS AND DISCUSSION

### The W195R mutation (EXOSC3 W238R) exhibits the most severe growth phenotype when introduced into Rrp40p

To gain further insights into the potential mechanisms by which EXOSC3 mutations cause PCH1B and related disorders, we introduced some of these mutations into the *S. cerevisiae* homolog of EXOSC3, Rrp40p. Among all the mutations implicated in these pathologies, we chose to study three mutations that affect residues conserved in *S. cerevisiae* Rrp40p (Fig. 1A): G31A, G191C, and W238R. This choice was based on the consideration that mutations that affect residues conserved throughout evolution are likely to affect fundamental aspects of the structure or function of the eukaryotic exosome. In addition, these mutations are associated with two distinct pathologies linked to EXOSC3 defects involving motor neurons (Halevy et al. 2014), suggesting that they may impact the function of the exosome in cerebellar and motor neurons in a fundamental manner. G31 (G8 in Rrp40p) resides in a hydrophobic pocket located near the interface with EXOSC5 (Rrp46p in yeast; Fig. 1B,C; Makino et al. 2013). Thus the G31A/G8A mutation might affect the interaction of EXOS3/Rrp40p with EXOSC5/Rrp46p (Fig. 1C) and thus the association of EXOSC3/Rrp40p with the exosome complex. W238 (W195 in *S. cerevisiae*) is located in the putative KH RNA binding domain of EXOSC3/Rrp40p (Fig. 1B), suggesting that mutations affecting this residue could impact RNA binding. In addition, W195 and G148 are located in close vicinity in the 3D structure of Rrp40p (Fig. 1D; Makino et al. 2013), suggesting that they might interact or cooperate to promote the structural or functional integrity of EXOSC3/Rrp40p and/or RNA binding (Fig. 1B).

**FIGURE 2.**
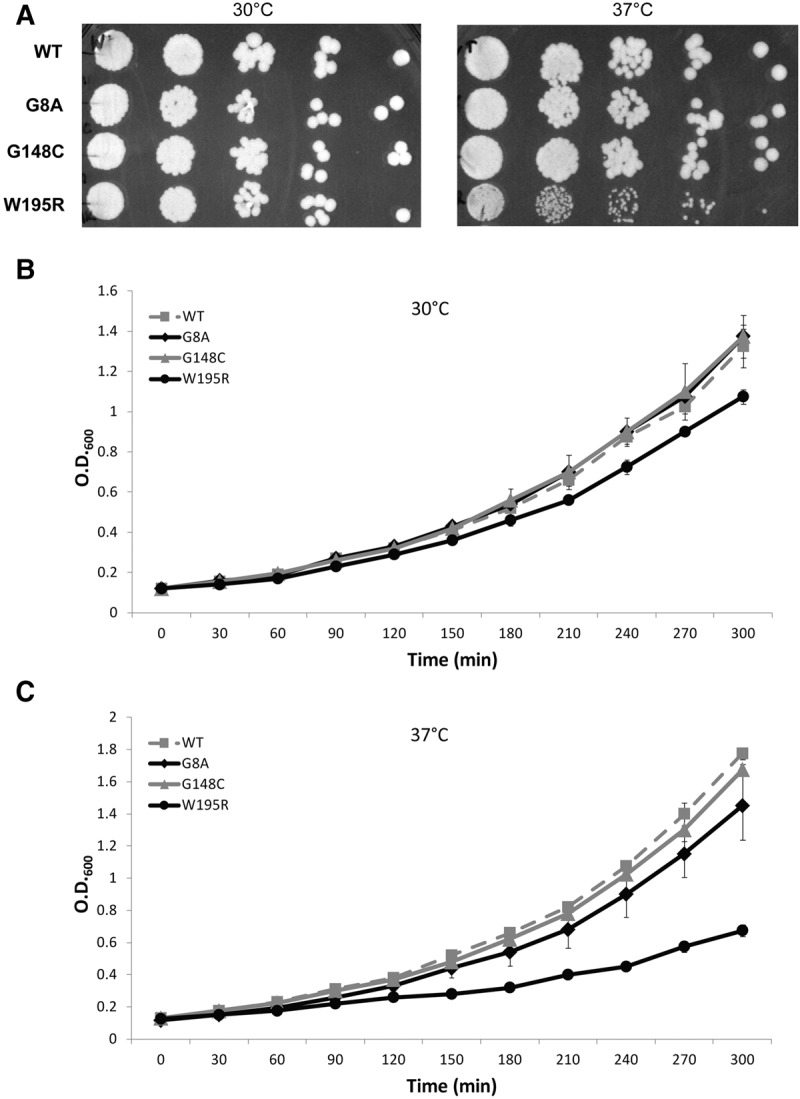
Growth of wild-type and Rrp40p mutants at 30° and 37°C. (*A*) Growth on solid medium at 30°C and 37°C. Serial dilutions of liquid cultures were spotted on YPD and grown at the indicated temperatures. (*B*) Growth on liquid medium at 30°C and 37°C. Shown are the average values and standard deviations observed for growth curves for two replicates performed simultaneously for each strain in the same batch of YPD medium and in the same incubator.

In order to avoid potential dosage effects, these three mutations were introduced by integration into the endogenous chromosomal copy of *RRP40* using the CRISPR system (Stovicek et al. 2015), rather than through extrachromosomal plasmid-borne copies. Accordingly, mutant versions of Rrp40p are expressed from the endogenous promoter and maintain identical 5′ and 3′UTR sequences. After introduction of each mutation at the chromosomal *RRP40* locus and confirmation by sequencing, growth of the mutant strains was compared to that of the isogenic wild-type strain at 30°C and 37°C, both in solid and liquid media. Analysis of the growth of these mutants on YPD plates by serial spot dilution showed no obvious growth defect on rich medium at 30°C, but the W195R mutation resulted in a detectable growth impairment at 37°C (Fig. 2A). In contrast, the G8A and G148C mutants did not exhibit detectable growth delay on solid medium. However, because cell density on solid media can mask subtle growth phenotypes, we also assessed mutant strains growth in liquid medium at both temperatures (Fig. 2B). Analysis of growth curves performed in simultaneous biological replicates performed in the same batch of medium and in the same incubator confirmed the severe growth defect of the W195R mutant at 37°C and also revealed a significant growth defect for the W195R mutant at 30°C compared to the wild-type strain. In addition, while the G8A and G148C mutants grew similarly to wild type at 30°C, their growth was clearly delayed compared to the wild-type reference strain at 37°C. Similar growth defects were observed in a separate independent experiment (Supplemental Fig. S1). In conclusion, all of the mutations studied here that result in PCH1b or hereditary spastic paraplegia cause detectable growth defects when transposed into yeast Rrp40p, with the W195R causing the most severe growth phenotype.

### Mutations of Rrp40p have a modest impact on snRNA processing and degradative functions of the exosome

**FIGURE 3.**
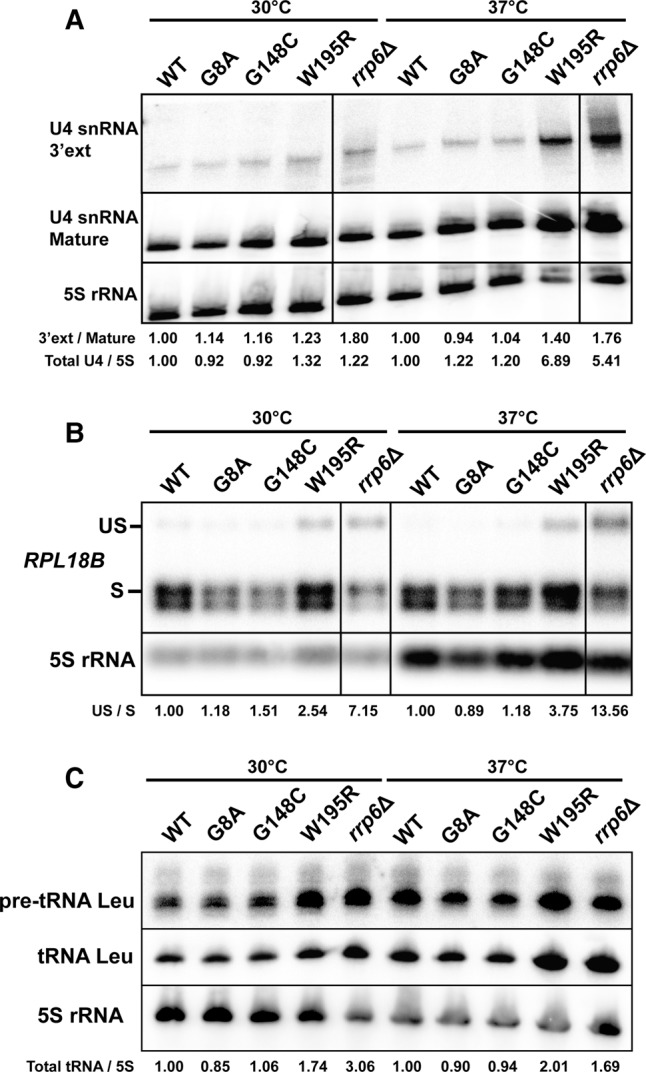
snRNA processing and degradation defects of Rrp40p mutants. (*A*) Analysis of U4 snRNA processing. Membranes were hybridized with an oligonucleotide probe downstream from the U4 snRNA or to an oligonucleotide hybridizing to the mature U4 snRNA. Lines drawn on the Northern blots indicate that lanes from these blots were removed from the picture. However, all the lanes shown for each panel originate from the same membranes and hybridizations. The 5S rRNA was used as a loading control. The values shown indicate the ratio of U4 3′-extended species to mature species, normalized to that ratio for the respective WT sample at each temperature. Also provided are the total U4 signals normalized to the 5S rRNA signals, which are in turn normalized to that ratio for the respective WT sample at each temperature. (*B*) Analysis of *RPL18B* spliced and unspliced RNA levels. The 5S rRNA was used as a loading control. Quantitation of unspliced to spliced (US/S) *RPL18B* was performed similarly to *A*. (*C*) Analysis of Leu pre-tRNA and tRNA levels. The 5S rRNA was used as a loading control. Quantitation of total tRNA signals was performed similarly to *A* and *B*.

To investigate the molecular effects of Rrp40p mutations, we chose to analyze a subset of exosome substrates by Northern blot analysis, as this technique provides the best way to specifically detect unprocessed species and intermediates, and to visualize the ratio of these species to mature RNAs. We chose to use as a loading control the 5S rRNA, since it was shown to be unaffected by various mutations affecting exosome function (Gudipati et al. 2012). The exosome is involved in several 3′-end processing steps during processing of the ribosomal RNA precursor and also processes the 3′-ends of several small nuclear RNAs (Allmang et al. 1999a; van Hoof et al. 2000). In addition, the exosome promotes the degradation of unspliced RNAs (Bousquet-Antonelli et al. 2000; Sayani and Chanfreau 2012) and of many precursor RNA species, including tRNAs (Allmang et al. 2000; Gudipati et al. 2012). Northern blot analysis of the U4 snRNA, whose precursor is cleaved by Rnt1p and then trimmed by the exosome (van Hoof et al. 2000), showed that the W195R mutation had the most severe impact on U4 snRNA processing, with an accumulation of the 3′-extended form of U4 at 37°C (Fig. 3A). Comparison with a sample generated from the Rrp6p knockout in which the 3′-extended form of U4 accumulates (Egecioglu et al. 2006) showed that this intermediate accumulated to levels slightly lower than in the sample from the *rrp6*Δ strain when strains were grown at 37°C, suggesting partial defects in the exosome function. In addition, we also detected an increase of the levels of mature U4 in the W195R and *rrp6*Δ strains (Fig. 3A). This increase might be due to reduced degradation of the U4 precursor by the exosome in these strains, as it was shown previously that the exosome degrades many precursor species of stable RNAs (Gudipati et al. 2012). The two other mutations did not exhibit any significant accumulation of 3′-extended U4 snRNA at any of the growth temperatures analyzed (Fig. 3A).

The nuclear exosome also degrades a large number of nuclear transcripts, including hypomodified tRNAs (Kadaba et al. 2004), CUTs, and many precursor species (Allmang et al. 2000; Gudipati et al. 2012). To investigate the impact of Rrp40p mutations on the exosome surveillance functions, we analyzed the accumulation of unspliced species of *RPL18B* and of Leu pre-tRNA, both of which were previously shown to be degraded by the exosome (Gudipati et al. 2012; Sayani and Chanfreau 2012). The W195R mutation exhibited an accumulation of unspliced *RPL18B* when this mutant was grown at either 30°C or 37°C, indicative of defective surveillance functions similar to or slightly milder than those detected in the *rrp6*Δ strain (Fig. 3B). Analysis of the Leu tRNA precursors and mature species also showed an accumulation of precursor species in the W195R mutant at 30°C and 37°C. At 37°C this mutant also exhibited elevated levels of mature Leu tRNA (Fig. 3C), comparable to those detected in the *rrp6*Δ strain. This result is consistent with previous observations showing that degradation of pre-tRNA precursors by the exosome titrate these species away from the processing pathway (Gudipati et al. 2012), and that perturbation of exosome function—either by Rrp6p inactivation, or by the W195R mutation—increases tRNA levels by redirecting more precursors into the processing pathway, in a manner similar to what we observed previously for U4 (Fig. 3A). The other two *RRP40* mutations did not exhibit any detectable phenotypes using these three exosome substrates, suggesting that their modest growth defect at 37°C (Fig. 2B) is due to other processing or degradation defects.

### Mutations of Rrp40p dramatically impair pre-rRNA processing

**FIGURE 4.**
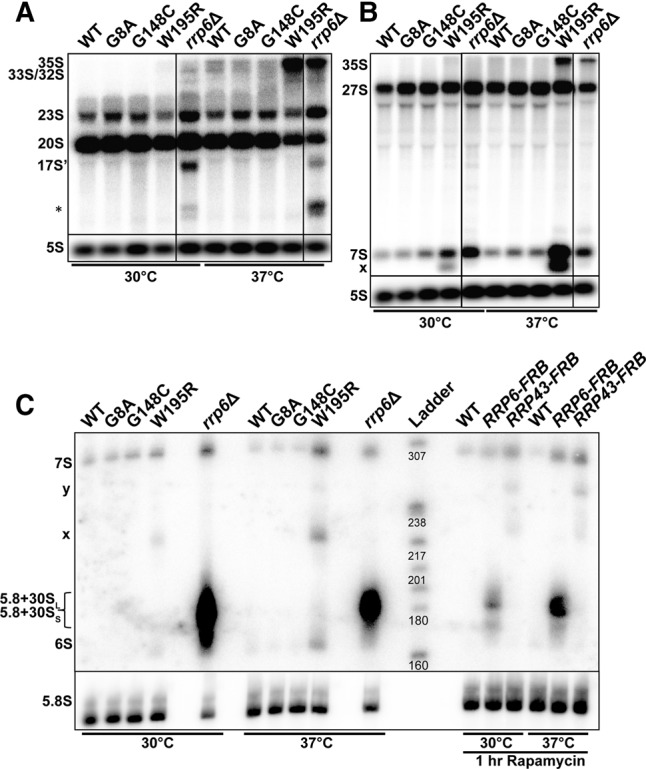
rRNA Processing analysis of Rrp40p mutants. (*A*) Agarose Northern blot analysis of precursor and intermediates containing the ITS1 sequence. Membranes were probed with probe O1663 (Supplemental Fig. S2) hybridizing to ITS1. The main precursors and intermediates containing the ITS1 sequence are labeled on the *left*. The species labeled 17S′ and (*) are species that are detected specifically in the *rrp6*Δ mutant strain (Wery et al. 2009). The latter species is not well characterized but has been detected previously (Wery et al. 2009). (*B*) Agarose Northern blot analysis of precursor and intermediates containing the ITS2 sequence. Membranes were probed with probe O1660 (Supplemental Fig. S2) hybridizing to the ITS2 spacer. The main precursors and intermediates containing the ITS2 sequence are labeled on the *left*. The species labeled with “x” are the ones that accumulate specifically in the W195R mutant. (*C*) Acrylamide Northern blot analysis of precursor and intermediates containing the ITS2 sequence. Membranes were probed with probe 020 hybridizing to the ITS2 spacer (Supplemental Fig. S2). The main precursors and intermediates containing the ITS2 sequence are labeled on the *left*. The species labeled with “x” are the same as the ones highlighted in panel *B*. The species labeled with “y” accumulates only in the W195R mutant and in the Rrp43 anchor away strain. The Rrp6 and Rrp43 anchor away strains and corresponding control strain were treated with Rapamycin to induce export of FRB-tagged proteins out of the nucleus.

We next analyzed the impact of the G8A, G148C, and W195R mutations on pre-rRNA processing by Northern blot analysis using probes hybridizing to both internal transcribed spacer (ITS1 and ITS2) sequences of the pre-rRNA precursor (Fig. 4A,B; Supplemental Fig. S2 shows a schematic representation of the pre-rRNA processing pathway with the location of the probes used). This analysis revealed that the W195R mutation resulted in a strong accumulation of the 35S precursor at 37°C (Fig. 4A,B). In addition, this mutant showed reduced accumulation of the 20S and 23S species at 37°C (Fig. 4A). Since the production of the 20S does not involve any exonuclease trimming (Supplemental Fig. S2), reduction of the levels of this intermediate in the W195R mutant is likely the result of an indirect effect and indicative of a general delay in pre-rRNA processing, consistent with pre-35S accumulation. The *rrp6*Δ strain showed the accumulation of 23S, 17S′, and (*) species (Fig. 4A; species described in Supplemental Fig. S2) reported previously (Wery et al. 2009). Interestingly, the G8A and G148C mutants exhibited increased levels of the 23S (Fig. 4A), indicative of defective processing or degradation of this intermediate. Hybridization with an ITS2 probe showed that the W195R mutation resulted in a severe accumulation of the 7S intermediate, particularly at 37°C, which reflects a delay in the processing leading to the production of the 5.8S rRNA (Fig. 4B). In addition to the accumulation of the 7S intermediate, we also detected the accumulation of faster migrating species, which might correspond to processing intermediates between the 7S and 5.8S rRNA (Fig. 4B). Interestingly, the G8A and G148C mutants also exhibited a slight but detectable accumulation of the 7S intermediate, especially at 37°C, indicative of a processing delay of the 5.8S rRNA in these mutants. This phenotype, combined with the 23S accumulation described above might be responsible for the slight growth defect detected in these mutants (Fig. 2B).

To gain a better resolution onto the processing defects of the 5.8S rRNA, we repeated this Northern blot analysis on an acrylamide gel, which allowed for a better separation of these species (Fig. 4C). This analysis confirmed the accumulation of the 7S intermediate in the W195R mutant, particularly at 37°C. In addition, it also showed the appearance of processing intermediates, which migrate between the 7S pre-rRNA and the 5.8S rRNA. These intermediates are distinct from the extended species of the 5.8S that accumulate in the *rrp6*Δ or Rrp6p-anchor away strains (marked as 5.8S + 30S/L on Fig. 4C) and correspond to the same species detected in strains in which core components of the exosome are inactivated, as shown by comparison with a strain in which Rrp43p is exported out of the nucleus (Fig. 4C). Rrp43p nuclear export was performed using the anchor away technique, by fusing Rrp43p to the FRB protein domain and treating cells with Rapamycin (Haruki et al. 2008). The pattern detected on Figure 4C was similar to the one observed when exosome subunits were depleted using conditional promoters (Allmang et al. 1999a). In conclusion, we found that all three Rrp40p mutations affect the pre-rRNA processing functions of the exosome, albeit to different extents and severity. Because the effects of the W195R mutation on rRNA processing are the most pronounced and correlate with the most severe growth defects, and because the molecular effects of the G8A and G148C mutants can only be detected on rRNA processing, we conclude that the most significant impact of the neurological mutations when introduced into Rrp40p is on the processing of the pre-rRNA precursor.

A recently published study also analyzed in *S. cerevisiae* the impact of some of the same mutations analyzed here (Fasken et al. 2016). Our results are consistent with most of the results reported in this study, including the effects on growth. Interestingly, this study showed that the W195R mutation results in an unstable protein, which does not associate well with the exosome complex and is degraded by the proteasome, especially when coexpressed with the wild-type version of Rrp40p (Fasken et al. 2016). This result suggests that the growth and RNA processing phenotypes of the W195R mutant are due to destabilization of the Rrp40p/EXOSC3 protein, leading to a fraction of the exosome complexes lacking this subunit, which might potentially destabilize the complex or lack the ability to associate with RNA (Fig. 1B).

One of the major differences between our results and those reported in this study is that the authors did not find any impact of these mutations on pre-rRNA processing, as no accumulation of the ITS-2 containing pre-rRNA species was detected by RT-PCR for the two mutants analyzed, G8A and W195R (Fasken et al. 2016). In contrast, our analysis clearly shows that some of the Rrp40p mutations significantly impact pre-rRNA processing, with a large accumulation of some precursors, intermediates, and aberrant forms for the W195R mutant, and a more subtle but detectable phenotype for the two other mutants (Fig. 4). We believe that this discrepancy may have been caused by the primer set used by Fasken et al. (2016) in their RT-PCR analysis. The reverse primer used in this study is positioned just downstream from the C2 cleavage site in the ITS2 (Supplemental Fig. S2) and is therefore not appropriate to amplify the 7S pre-rRNA and shorter processing products (we also note that there is a switch between the forward and reverse primers in Supplemental Table 2). This difference is sufficient to explain the fact that Fasken et al. (2016) did not detect an accumulation of the 7S species in their mutants, leading to a similar ITS2 signal for all strains analyzed.

The findings that we describe here in yeast are reminiscent of the clinical manifestations in patients who harbor different mutations affecting the *EXOSC3* gene. Among the three mutations tested, the W238R (*S. cerevisiae* W195R) is associated with the most severe clinical phenotype, correlating with the severe growth defect described here in yeast. This genotype was found in a sib pair compound heterozygous for the G31A substitution, presenting with congenital profound cerebellar hypoplasia with hypotonia and global developmental delay, dying by age 7–8 mo (Wan et al. 2012; Rudnik-Schöneborn et al. 2013). The G31A mutation (c.92G > C genotype) was first discovered in patients who presented with congenital cerebellar hypoplasia with spinal motor neuron involvement and death during infancy (Wan et al. 2012). This mutation was subsequently identified in additional patients of Czech Roma origin (Schwabova et al. 2013; Eggens et al. 2014). Patients homozygous for the c.92G > C;p.G31A mutation rarely survive infancy, with survival ranging up to 17 mo (Schwabova et al. 2013; Eggens et al. 2014). Finally, the G191C mutation (genotype c.571G > T) was associated with the mildest clinical features with the least cognitive impairment and the longest survival. Homozygous c.571G > T;p.Gly191Cys was identified in two sib pairs in a consanguineous family who presented with progressive spastic paraplegia of childhood onset, reflecting upper motor neuron involvement with survival into adulthood in the setting of mild cerebellar hypoplasia without spinal motor neuron disease (Halevy et al. 2014).

Interestingly, analysis of 5.8S rRNA processing in human fibroblasts from a subject in a family carrying a D132A mutation in EXOSC3 did not reveal any significant accumulation of the 7S intermediate (Wan et al. 2012). It is possible that this mutation, which correlates with a relatively mild phenotype, might not be sufficiently damaging to result in any processing defect in normal conditions. Based on the results presented in this study and on the similarities between the severity of the phenotypes of the mutations in Rrp40p and EXOSC3 and on this previous analysis, we propose that mutations of EXOSC3 that result in neurological diseases affect rRNA processing and that the severity of processing defects is correlated with that of the growth defects in yeast and of the pathology in human patients. It remains to be established why cerebellar and motor neurons are particularly prone to defects in exosome function.

## MATERIALS AND METHODS

Exosome mutants were generated using the CRISPRm system in a BY4742 background strain. pCAS plasmids containing specific sgRNAs proximal to each missense mutation were constructed using phosphorylated primers, as previously described (Stovicek et al. 2015). BY4742 cells were transformed with 1 µg of pCAS plasmid and 5µg of 60-mer homologous DNA harboring the desired missense mutations as previously described (Ryan et al. 2014). Transformants were plated on YPD + G418 and grown at 37°C for two nights and then transferred to 30°C. Individual colonies were patched on YPD plates and checked by PCR and sequencing. For liquid culture growth, yeast strains were grown to exponential phase in YPD and then back-diluted in YPD to OD = 0.10 at *t* = 0. Cell densities were measured approximately every 30 min thereafter for 3 h. Northern blot analysis and quantitation of signals were performed as described in Gabunilas and Chanfreau (2016). Most Northern blots were hybridized with 5′-end labeled oligonucleotide probes listed below, with the exception of the RPL18B blots, which were hybridized with an antisense RNA probe generated from a PCR product obtained with the RPL18B T3 FW/RV primers listed below.

U4 3′ ext: CAGTCCCTTTGAAAGAATGAAT
U4 mature: CGGACGAATCCTCACTGATA
tRNA Leu mature: GGCGCCTGATTCAAGCTCAGGTATCGTAAG
tRNA Leu intron-mature hybrid probe: CGATACCTGAGTATTCCCAC
RPL18B T3 Fwd: TATTAGGTTGGGAAAGAGGG
RPL18B T3 Rev: AATTAACCCTCACTAAAGGGAGGCACCAAAGGTATATGGTGTGGG
1663 (ITS1): CTCTTGTCTTCTTGCCCAGTAAAAG
1660 (ITS2): AGGCCAGCAATTTCAAGTTAACTCC
020 (ITS2): TGAGAAGGAAATGACGCT
5.8S rRNA Probe: CCAAGGGGCGCAATGTGCGTTCAAAGATTCGATGA
5S rRNA Probe: CTCGGTCAGGCTCTTACCAGCTTAACTACA

## SUPPLEMENTAL MATERIAL

Supplemental material is available for this article.

## Supplementary Material

Supplemental Material
