# Supplementary material for: Mutations of EXOSC3/Rrp40p associated with neurological diseases impact ribosomal RNA processing functions of the exosome in S. cerevisiae
Source: RNA. 2017 Apr;23(4):466–72. doi: 10.1261/rna.060004.116 (PMC5340910; doi:10.1261/rna.060004.116)
Supplement: Supplemental Material [file supp_23_4_466__index.html]

Mutations of EXOSC3/Rrp40p associated with neurological diseases impact ribosomal RNA processing functions of the exosome in S. cerevisiae — Mutations of EXOSC3/Rrp40p associated with neurological diseases impact ribosomal RNA processing functions of the exosome in S. cerevisiae — Supplemental Material 

# Mutations of EXOSC3/Rrp40p associated with neurological diseases impact ribosomal RNA processing functions of the exosome in *S. cerevisiae*

## Supplemental Material

**Files in this Data Supplement:**

- Supplemental Figure S1.tif
- Supplemental Figure S2.tif
