# Supplementary figures and images for: Mutations of EXOSC3/Rrp40p associated with neurological diseases impact ribosomal RNA processing functions of the exosome in S. cerevisiae
Source: RNA. 2017 Apr;23(4):466–72. doi: 10.1261/rna.060004.116 (PMC5340910; doi:10.1261/rna.060004.116)

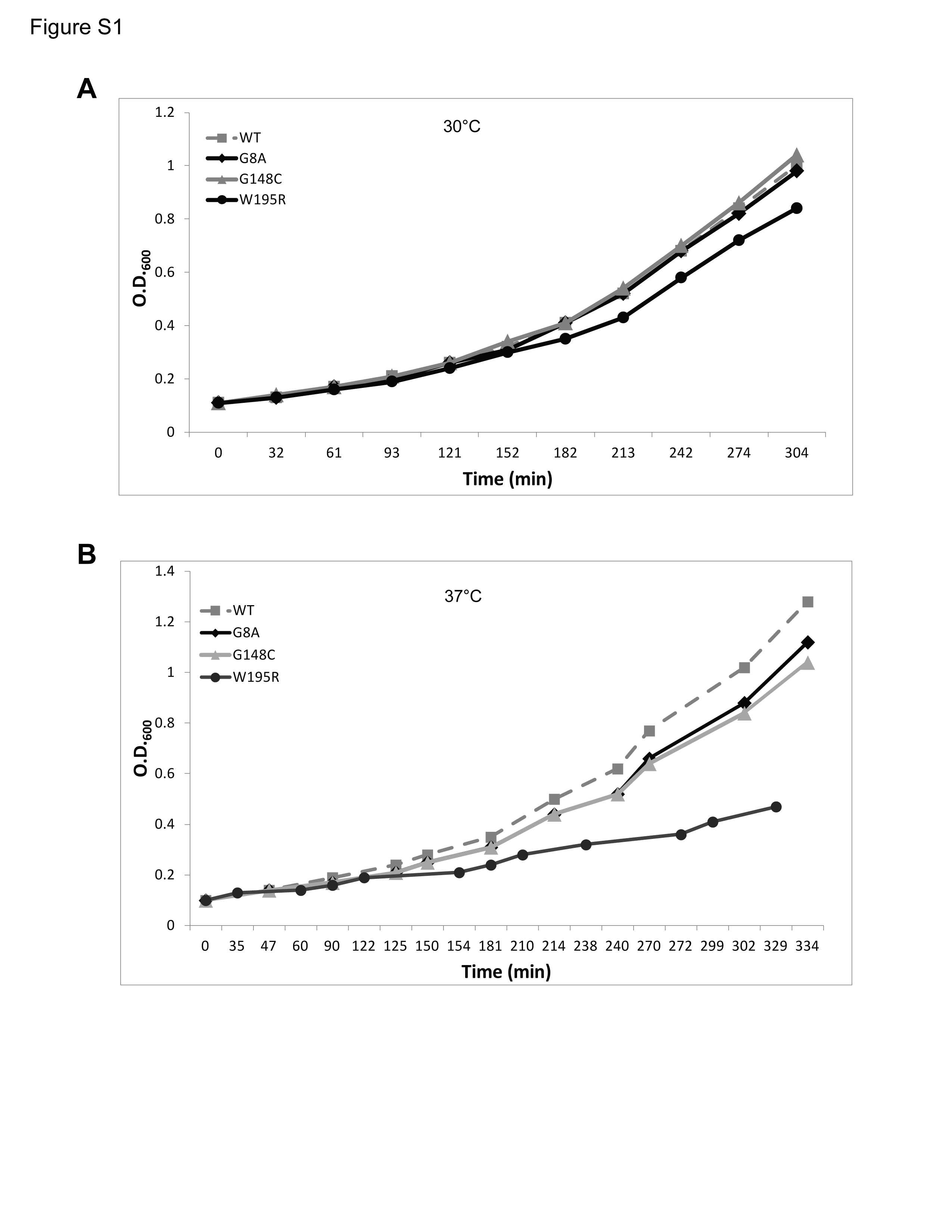

Supplement: Supplemental Material [file supp_060004.116_Supplemental_Figure_S1.tif]

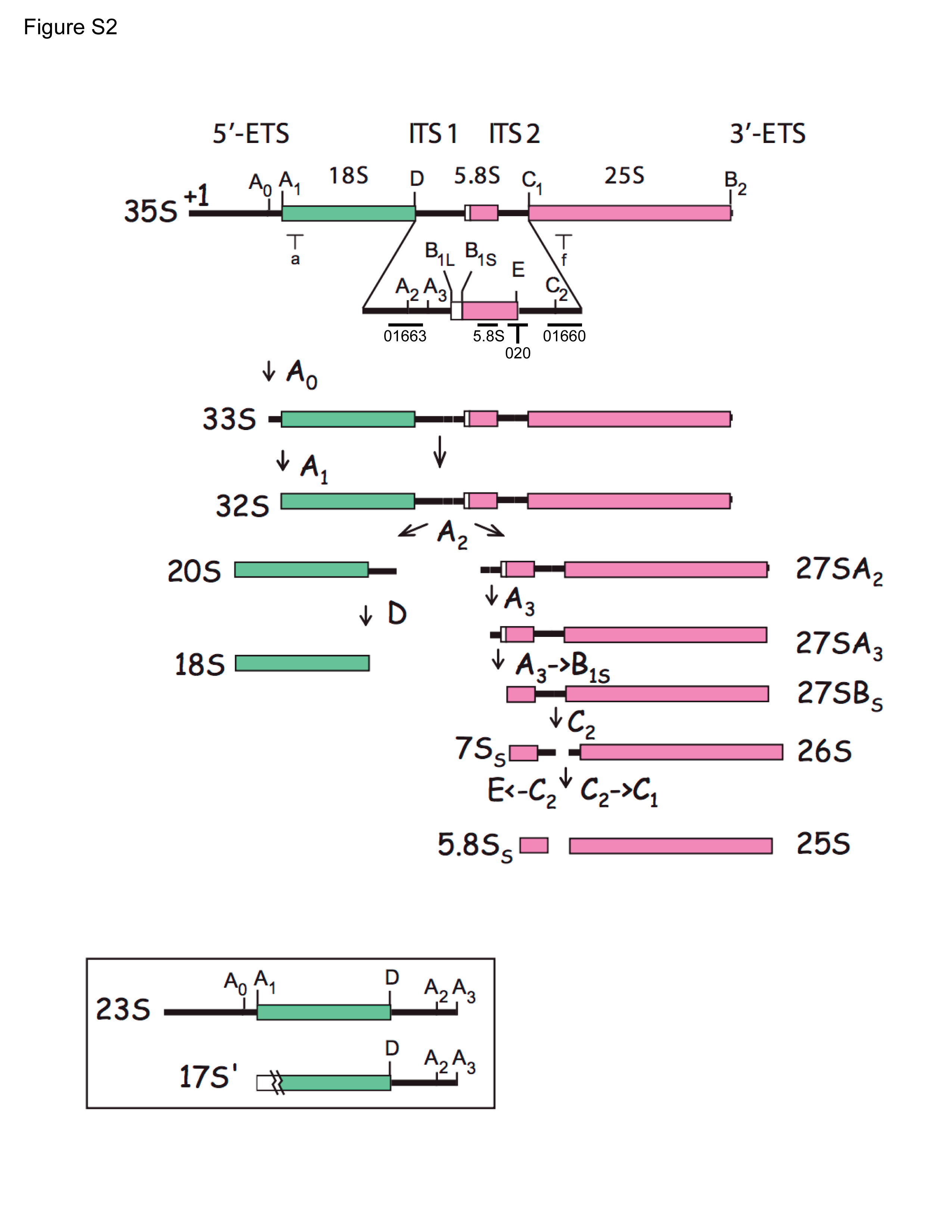

Supplement: Supplemental Material [file supp_060004.116_Supplemental_Figure_S2.tif]
